# Food and governance practices in schools: when health becomes a corporate issue

**DOI:** 10.1590/0102-311XEN025324

**Published:** 2024-04-29

**Authors:** Pablo Piquinela

**Affiliations:** 1 Facultad de Psicología, Universidad de la República, Montevideo, Uruguay.; 2 Instituto de Investigaciones Gino Germani, Universidad de Buenos Aires, Buenos Aires, Argentina.

The book *School Food Politics in Mexico: The Corporatization of Obesity and Healthy Eating Policies* ^1^ was written by José Tenorio and published by Routledge in 2024. It presents an articulation of the historical, political, and cultural dimensions of food based on an analysis of public policies and the governance of food practices in Mexico.

The study is framed within a broad and growing field of food studies, nourished by analyses from anthropology and sociology ^2^ ^,^ ^3^ ^,^ ^4^ ^,^ ^5^ ^,^ ^6^. The work contributes to the field of study that looks into diet as a composition that can be analyzed from different dimensions, such as political, social, economic, among others. The study presented in the book aims to build tools that allow the reader to think about diet in an alternative way to the rationality that configures food practice as an individual and rational decision that serves as a basis for the design of public policies and nutritional and psychological interventions.

There are many discourses on how to lead a healthy life. In this sense, the text, based on a case study of Nestlé in Mexico, makes a substantial contribution to thinking about the productive role of corporations in shaping the obesity epidemic as an artifact. Such disposition has been identified by several authors as an attempt to depoliticize and decontextualize a problem that is presented as entangled ^7^ and that, as posed by corporations, is purposed to reinforce a mode of subjectivation that aims to increase human capital and make people morally responsible for leading a healthy life ^8^ ^,^ ^9^.

Tenorio uses Michel Foucault’s tool of the *dispositif* as a method of analysis that takes into account the network that can be established between these elements ^10^. Under these coordinates, the book seeks to describe and analyze the arrangement of an amalgam of discourses, knowledge, programs and objects that are arranged to constitute the so-called healthy lifestyles of policies and programs: dishes and posters in schools, the role of teachers, cooks, researchers and corporations are part of an amalgam in which food becomes an edible object linked to the health and life of the population.

If we consider its strategic function, i.e., the fact that each device arises from a social or historical problem that is urgent, Tenorio shows how the “fight” against obesity is beginning to be the political problem to which government efforts should be directed. The book shows how the lines of knowledge that support the problem gain consistency. Tenorio critically analyzes the model of the energy balance, the model of individual responsibility in food choices and that of multifactorial discourses. In this field of interactions, the relationship of power is inseparable from the forms of knowledge, meaning that it can be reversed or inverted ^11^. In this key, and thinking about its contribution in methodological terms, ethnographic fieldwork provides very valuable and unique information to understand the meaning of food practices and the polysemic drifts that policies acquire in their implementation.

In order to think about these dispositions, the author carries out a theoretical-empirical analysis that focuses on three dimensions: (i) a historical analysis of the process of liberalization of the Mexican economy in relation to the process of neoliberalization of diets; (ii) the discursive practices of healthy eating policies and their promoters and executors - food corporations -, from the critical analysis of the healthy syntagma; and (iii) the polyvalence acquired by food practices in the territorial execution of these policies, which allows the author to understand the lines of resistance presented by the device, based on ethnographic fieldwork from which he gets to know and interpret the practices of educators, cooks and children.

The book is divided into seven chapters. It constructs a research problem that investigates issues such as the emergence of this idea, the type of subjects it assumes, the practices it promotes and those it does not, and seeks to open the discussion on obesity and the search for a healthy life to aspects from which it is usually separated, such as the political, economic and cultural ones ^1^. The introductory chapter (*Governing Through Healthy Lifestyles*) shows how obesity has been constituted as a problem and as an apparatus of healthy living. Chapter 2 (*Food, Public Health and Education in the Making of Mexico*) analyzes how the school environment has become a privileged space to regulate eating practices and how it has generated efforts to be transformed from an “obesogenic environment” to an “anti-obesity site”. Chapter 3 (*The Cultural Politics of Language in Obesity Policy*) presents a study of public policies against obesity in Mexico based on documents and interviews with implementers. The section is oriented towards thinking about the multifactorial nature of obesity as a rationality that supports policies, in order to analyze what and how are the forces and regimes of knowledge that support the promotion of healthy lifestyles. In Chapter 4 (*Corporatizing Healthy Eating*), the case of the Mexican state of Veracruz and its anti-obesity strategy is presented. The chapter shows how Nestlé, using its corporate social responsibility (CSR) programs, plays a role in building campaigns that appeal to shared responsibility and how data collection practices support policies aimed at strengthening people’s decision-making capacity. The next chapter (*Beyond Policy. Everyday Cooking in Schools*) analyzes ethnographic encounters in the Benito Juárez and Emilio Zapata schools. Tenorio works on what he considers to be the forgotten aspects of the political narrative that makes schools the ideal spaces for “fighting” obesity with solutions that, decontextualized, are supported by discourses of energy balance and shared responsibility. At the same time, the ethnographic work allows him to explain the meanings that cooks give to food and to the different possible combinations, which are the signs that can be understood as resistance in the practices of the kitchens of these schools. In *Healthy Lifestyles, Bottled Water and Corporate Profits*, chapter 6 of the book, Tenorio takes water consumption as a case of analysis, showing that policies and programs aimed at fighting obesity have been partially successful in reducing the consumption of sugary drinks in schools, but the increase in water consumption, due to the lack of confidence in tap water and the absence of available taps with drinking water for public access, has increased corporate profits based on the sale of bottled water in smaller containers designed for consumption outside the home. Lastly, the *Food Through Schools: What Futures?* section proposes moving from policies based on empowerment and behavior change to the creation of a national school lunch program that provides schools with the resources they need to adequately feed students.

The book describes how a new production model based on the Green Revolution has led to a food transition in which problems of scarcity have been compounded by problems of excess. The research could be extended to include the dimension of the body in the problem, raising some initial concerns: if this identified amalgam aims to build anti-obesity sites, that is, to prioritize the bodies that are considered healthy, what are the implications for cooks, teachers and students? Likewise, considering that education circulates among multiple spaces, questions about where children obtain information about food, what resources and media transmit dietary discourses and what messages they convey, among other aspects, could serve as references to expand and continue this contribution by Tenorio, which undoubtedly makes an important contribution to the field of study.

## References

[1] Tenorio J (2024). School food politics in Mexico: the corporization of obesity and health eating policies..

[2] Pollan M (2006). The omnivore's dilemma: the search for a perfect meal in a fast-food world..

[3] Holt-Giménez E (2018). El capitalismo también entra por la boca: comprendamos la economía política de nuestra comida.

[4] Mahoney C (2015). Health, food and social inequality: critical perspectives on the supply and marketing of food..

[5] Sammartino G (2014). Notas para identificar el modelo de producción agroalimentario hegemónico actual. Diaeta.

[6] Aguirre P (2017). Una historia social de la comida.

[7] Guthman J (2011). Weighing in: obesity, food justice, and the limits of capitalism.

[8] Costa F, Costa F, Rodríguez P (2017). La salud inalcanzable. Biopolítica molecular y medicalización de la vida cotidiana..

[9] Sibilia P (2005). El hombre postorgánico. Cuerpo, subjetividad y tecnologías digitales..

[10] Foucault M (1991). Saber y verdad.

[11] Foucault M (1995). ¿Qué es la crítica?. Revista de Filosofía Daimon Internacional.

